# Accelerated repair of demyelinated CNS lesions in the absence of non-muscle myosin IIB

**DOI:** 10.1002/glia.22627

**Published:** 2014-01-28

**Authors:** Tomasz Rusielewicz, Jennifer Nam, Evangelos Damanakis, Gareth R John, Cedric S Raine, Carmen V Melendez-Vasquez

**Affiliations:** 1 Department of Biological Sciences, Hunter College, New YorkNew York; 2 The Graduate Center Molecular Cellular and Developmental Biology, The City University of New YorkNew York; 3 Corinne Goldsmith Dickinson Center for Multiple Sclerosis and Department of Neurology, Mount Sinai School of Medicine, New YorkNew York; 4 Department of Pathology, Albert Einstein College of MedicineBronx, New York; 5 Department of Neurology, Albert Einstein College of MedicineBronx, New York; 6 Department of Neuroscience, Albert Einstein College of MedicineBronx, New York

**Keywords:** oligodendrocytes, remyelination, cytoskeleton, myosin II

## Abstract

The oligodendrocyte (OL), the myelinating cell of the central nervous system, undergoes dramatic changes in the organization of its cytoskeleton as it differentiates from a precursor (oligodendrocyte precursor cells) to a myelin-forming cell. These changes include an increase in its branching cell processes, a phenomenon necessary for OL to myelinate multiple axon segments. We have previously shown that levels and activity of non-muscle myosin II (NMII), a regulator of cytoskeletal contractility, decrease as a function of differentiation and that inhibition of NMII increases branching and myelination of OL in coculture with neurons. We have also found that mixed glial cell cultures derived from NMIIB knockout mice display an increase in mature myelin basic protein-expressing OL compared with wild-type cultures. We have now extended our studies to investigate the role of NMIIB ablation on myelin repair following focal demyelination by lysolecithin. To this end, we generated an oligodendrocyte-specific inducible knockout model using a Plp-driven promoter in combination with a temporally activated CRE-ER fusion protein. Our data indicate that conditional ablation of NMII in adult mouse brain, expedites lesion resolution and remyelination by Plp+ oligodendrocyte-lineage cells when compared with that observed in control brains. Taken together, these data validate the function of NMII as that of a negative regulator of OL myelination *in vivo* and provide a novel target for promoting myelin repair in conditions such as multiple sclerosis.

## Introduction

The process of myelination, which provides neuronal axons with a specialized proteolipid-rich membrane, permits rapid conduction of action potentials and is orchestrated in the central nervous system (CNS) by a subset of glial cells called oligodendrocytes (OL). Although myelin is not necessary for neuronal communication at short distances, it becomes vital when connecting processes of greater length, such as those between the two hemispheres of the brain or the length of the spinal cord. Aside from its electrical insulator properties, myelin also plays a role in axonal maintenance [reviewed in Nave (2010)]. Loss of myelin in diseases such as multiple sclerosis (MS) results in conduction failure, and underlies many of the clinical deficits (weakness, paralysis, and blindness) associated with this disorder.

MS is believed to be an autoimmune disorder of the human CNS, with inflammatory cells invariably present in demyelinating lesions, but the primary cause of lesion formation and whether it originates from a problem with myelin itself or some environmental insult, is not well understood. Recovery of neural function accompanies remyelination, which restores nerve conduction and re-establishes the normal molecular organization of the myelinated axon (Salzer, 2003). Remyelination is mediated by several sources of oligodendrocyte precursor cells (OPC), some of which like neural progenitors cells migrate from the sub-ventricular zone, while others such as NG2+ progenitors are found throughout the adult CNS (Levine et al., 2001; Nielsen et al., 2006). Although remyelination in MS is initially associated with OPC proliferation (Raine et al., 1981), over time as lesions expand and become chronic, this repair process becomes less efficient, as there is little that can be done to limit the progressive burden on the reservoir of OPC resulting from recurrent demyelination (Franklin, 2002).

The cellular mechanism whereby OL extend multiple cell processes that wrap around CNS axons is still poorly defined but involves active remodeling of the cytoskeleton. These morphological changes have been linked to differentiation and myelin formation both *in vivo* and *in vitro* (Bauer et al., 2009; Kim et al., 2006; Sloane and Vartanian, 2007). We have previously shown that levels of non-muscle myosin II (NMII), which generates the force for cytoskeletal contractility, decrease as a function of OL differentiation and that inhibition of myosin activity increases branching and myelination by OL in co-culture with neurons (Wang et al., 2008). Our group has also demonstrated accelerated maturation of OL purified from NMIIB null mice (Wang et al., 2012).

As the process of remyelination recapitulates events taking place during normal OL development (Fancy et al., 2011; Moll et al., 2013), we have tested the hypothesis that conditional ablation of NMIIB in adult brain may promote myelin repair via acceleration of OL differentiation. Using the lysolecithin model of demyelination, we show here that targeted deletion of NMIIB from OL expressing proteolipid protein (Plp) accelerates lesion resolution and increases the number of mature CC1+ OL found inside the remyelinating lesion. Collectively, our results provide a novel strategy to enhanced myelin repair by promoting OL maturation.

## Materials and Methods

### Mice

*NMIIB ^fl/fl^* mice (Ma et al., 2009) were provided by Mary Anne Conti and Robert S. Adelstein (Laboratory of Molecular Cardiology, NHLBI) and are available from MMRRC (Stock #016981-UNC). *PlpCre/ESR1* mice (Doerflinger et al., 2003) were purchased from the Jackson Laboratory (Stock # 005975). Gt(ROSA)26Sortm4*(ACTB-tdTomato,-EGFP)Luo*/J (Rosa26-mT/mG) mice (Muzumdar et al., 2007) (Jackson Laboratory, Stock # 007576) were provided by Paul Feinstein (Hunter College, CUNY). *PlpCre/ESR1* hemizygous mice were crossed with Rosa26-mT/mG heterozygotes to check for recombination efficiency. *PlpCre/ESR1* males were crossed with *NMIIB ^fl/fl^* females and the F1 *PlpCre/ESR1*:*NMIIB ^fl/+^* males were then backcrossed to *NMIIB ^fl/fl^* females to generate *PlpCre/ESR1*:*NMIIB ^fl/fl^* (cKO) and NonCre:*NMIIB ^fl/fl^* (Control) mice for remyelination analysis. *PlpCre/ESR1*:*NMIIB ^fl/+^* animals were also crossed to the offspring (F1) from a Rosa26-mT/mG x *NMIIB ^fl/fl^* cross to generate *PlpCre/ESR1*:Rosa26-mT/mG:*NMIIB ^fl/fl^* (mT/mG; cKO) and *PlpCre/ESR1*:Rosa26-mT/mG:*NMIIB ^+/^^+^* (mT/mG; Control). All procedures were performed in accordance with the National Institutes of Health guidelines and were approved by Hunter College Institutional Animal Care and Use Committee.

### Tamoxifen Recombination and Lysolecithin Injections

Eight-week-old mice were injected intraperitoneally with 49 mg/kg tamoxifen for 5 consecutive days. For demyelination, 12-week-old mice injected unilaterally into the corpus callosum (5.5 mm anterior to lambda, 1 mm lateral to bregma, 2 mm deep) with 1.5 µL of a solution of 1% lysolecithin in PBS (Nait-Oumesmar et al., 1999). Animals were sacrificed 7, 14, or 28 days later. These time points correspond to well-characterized phases of active demyelination, proliferation and remyelination of lysolecythin-induced lesions (Zhang et al., 2009).

### Immunocytochemistry

Mice were euthanized and perfused transcardially with 4% paraformaldehyde. Brain-frozen sections (30 µm) were collected over a 1mm distance centered on the needle track. Sections were processed for immunofluorescence as described (Wang et al., 2008) and imaged using a Zeiss LSM 510 confocal microscope.

### Image Analysis

Image analysis was performed using ImageJ 1.46m and Adobe Photoshop CS5. Adjustment of image brightness or contrast was performed in some cases but without misrepresenting data. Lesion area was measured using the freehand selection tool inside FluoroMyelin negative areas. Lesion size and volume calculations were based on the number of sections collected for each lesion multiplied by their thickness and their average lesion area. Fluorescence intensity for specific antibodies within the lesion area was measured using the mean gray value. All measurements were normalized by the background mean gray value obtained from a section of normal appearing white matter adjacent to the lesion. When calculating remyelination by EGFP^+^ oligodendrocytes at 28 dpi, the particle analysis tool was used to measure the total cell area (including cell body and processes) within the lesion shadow (EGFP^−^, MBP^+^). This value was then divided by the total shadow area, to obtain the percentage covered by EGFP^+^ oligodendrocytes. Statistical tests were performed using Graph Pad Prism software.

### Histopathology

Mice were perfused with 2.5% gluteraldehyde and brain tissue was trimmed and postfixed in 1.5% osmium tetroxide followed by dehydration in 30–100% ethanol and embedding in Epon. Semithin sections (1 µm) were stained with toluidine blue and imaged using a Zeiss Axioplan microscope. For EM sections were stained with uranyl acetate and lead citrate and imaged using TEM (JEOL JEM-100C/HS-600SCX). *g*-Ratios [(axon diameter)/(fiber diameter)] were measured from electron micrographs of the corpus callosum of 16-week-old mice using Image J.

### Antibodies

NG2 (AB5320), CC1 (OP80), and Olig2 (AB9610) from EMD Millipore Corporation, Billerica, MA; GFAP (#3670, Cell Signaling Technology); NF (PCK-593P) and MBP (SMI-94R) from Covance; and Iba1 (#019-19741, Wako). FITC, Rho, or Cy5-conjugated secondary antibodies were purchased from Jackson ImmunoResearch Laboratories. FluoroMyelin (F34651) and Topro 3 (T3604) from Invitrogen were also used.

## Results

### Ablation of NMII in Adult Oligodendrocytes Does Not Affect Myelin Morphology

To explore the functional relevance of NMIIB to myelin repair *in vivo*, we generated mice with tamoxifen-inducible inactivation of NMIIB in glia, by mating *PlpCre/ESR1* mice (Doerflinger et al., 2003) to floxed NMIIB animals (*NMIIB ^fl/fl^*) (Ma et al., 2009). *PlpCre/ESR1:NMIIB ^fl/fl^* (cKO) mice are viable, phenotypically normal, and born at the expected Mendelian ratios. *PlpCre/ESR1* system has been extensively used to generate tamoxifen-induced, Cre-mediated recombination under the control of the myelin proteolipid protein (*Plp1*) promoter (Doerflinger et al., 2003; Forrest et al., 2009; Pillai et al., 2009); and previous studies have shown that upon treatment with tamoxifen, efficient recombination occurs in developing OPC and mature myelinating OL throughout the adult CNS as well as developing and mature Schwann cells in the peripheral nerve (Doerflinger et al., 2003; Leone et al., 2003).

**Figure 1:**
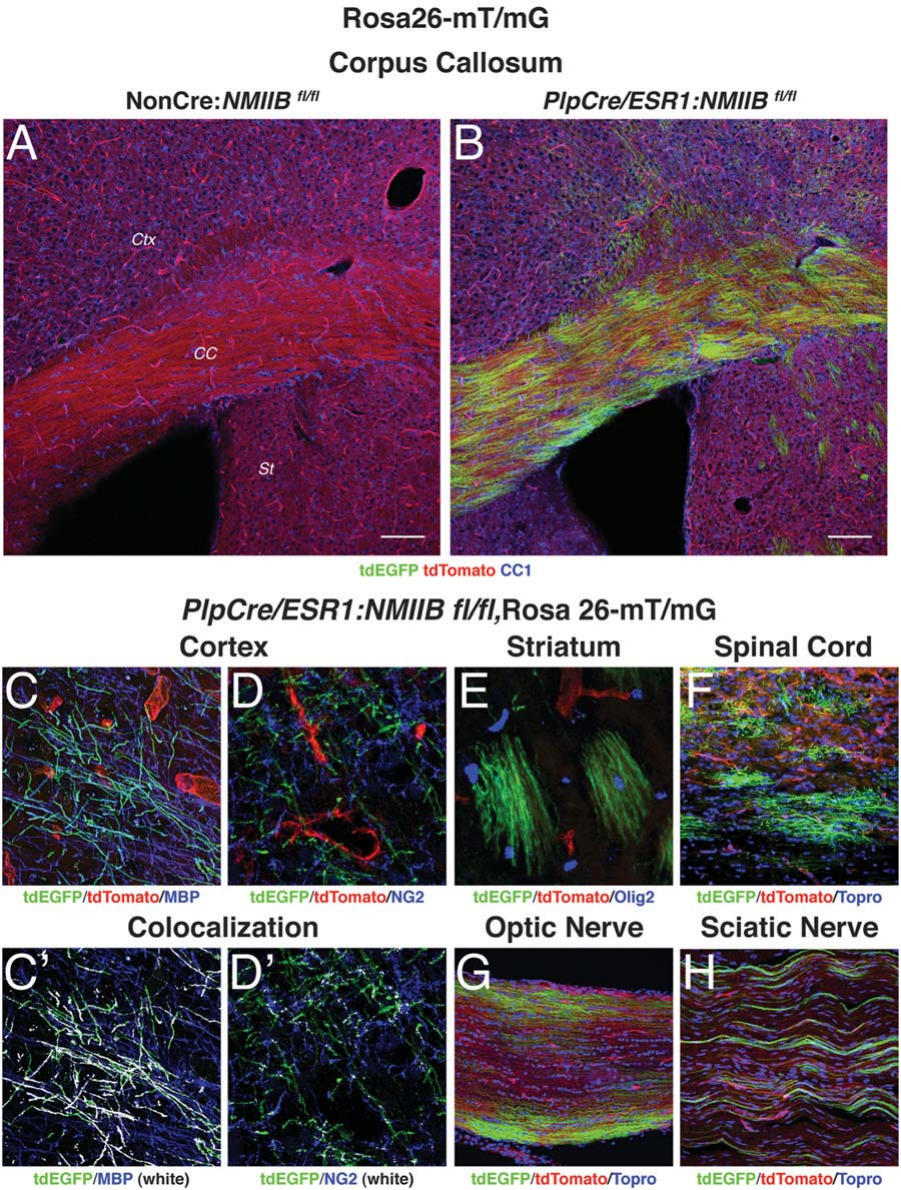
Analysis of tamoxifen-driven *PlpCre/ESR1* recombination. Rosa26-mT/mG mice have loxP sites on either side of a membrane-targeted tdTomato cassette and express strong red fluorescence in all tissues. When bred to *PlpCre/ESR1* mice, the resulting offspring have the mT cassette deleted in the Cre-expressing cells, allowing expression of the membrane-targeted EGFP cassette. *NMIIB ^fl/fl^* mice carrying both the *PlpCre/ESR1* transgene and the Rosa26-mT/mG allele (Plp-Cre^+^) or Rosa26-mT/mG allele alone (NonCre) were injected with tamoxifen for 5 days at 8 weeks of age. The animals were sacrificed at 12 weeks and their tissue was processed for immunofluorescence with antibodies to CC1, MBP, NG2, Olig2, and the nuclear stain Topro3. (A, B): Sections of the corpus callosum (CC) show high EGFP expression in OLs in the white matter tract as well as in the cerebral cortex (CTX) and striatum (St). Scale bars 100 µm. (C–H): Details of cells expressing EGFP throughout the CNS. In the cortex (C, D) EGFP label colocalizes (shown in white) with mature OL markers such as MBP (C′), but not with NG2 (D′). Recombination is also observed in myelinating OL in the striatum (E), spinal cord (F) and optic nerve (G) as well as in myelinating Schwann cells in the sciatic nerve (H).

We performed our own assessment of *PlpCre/ESR1* mediated-recombination 4 weeks after tamoxifen treatment of 8-week-old mice, using the reporter line Rosa26-mT/mG (Muzumdar et al., 2007) and confirmed extensive and efficient recombination in myelinating OL (MBP+, CC1+) throughout corpus callosum, cortex, striatum, spinal cord, and optic nerve (Fig. 1) as well as NG2- (Fig. 1D) cell with a morphology consistent with that of non-myelinating pre-oligodendrocytes (Chang et al., 2002). Approximately 87% of the MBP^+^ white matter area in the corpus callosum of 12-week-old cKO mice contained EGFP+ cells indicating extensive recombination. Recombination was also detected in myelinating Schwann cells in the sciatic nerve (Fig. 1H).

**Figure 2:**
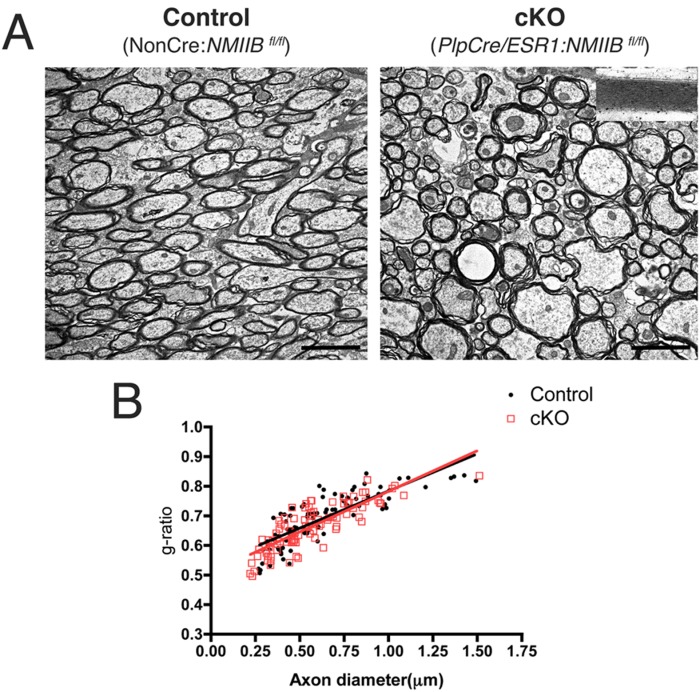
Morphological analysis of uninjured corpus callosum in control and cKO brains. (A): Electron micrographs from uninjured corpus callosum of control and cKO of 16-week-old mice reveal normal myelin morphology, periodicity (inset), and *g*-ratio (B). Values in graph were obtained from three animals per genotype, for a total of 183 axons measured. [Color figure can be viewed in the online issue, which is available at wileyonlinelibrary.com.]

NMIIB is the main myosin II isoform expressed by oligodendrocytes *in vitro* (Wang et al., 2012), and although their expression levels correlate negatively with myelination (Dugas et al., 2006; Wang et al., 2008), we evaluated whether conditional ablation of NMIIB in adult OL *per se* had an effect on myelin maintenance and/or organization. To this end, we examined uninjured brain from cKO (*PlpCre/ESR1:NMIIB ^fl/fl^*) and control (NonCre*:NMIIB ^fl/fl^*) mice by light and electron microscopy and confirmed the presence of normal appearing white matter in cKO brain (Fig. 2). Detailed morphometric analysis performed in 16-week-old mice (the end-point of our remyelination studies) showed no differences in *g*-ratio, myelin thickness, and/or periodicity between uninjured control and cKO brains. These results indicate that NMIIB expression is not required for myelin maintenance.

### Remyelination Is Accelerated in the Absence of NMII

**Figure 3:**
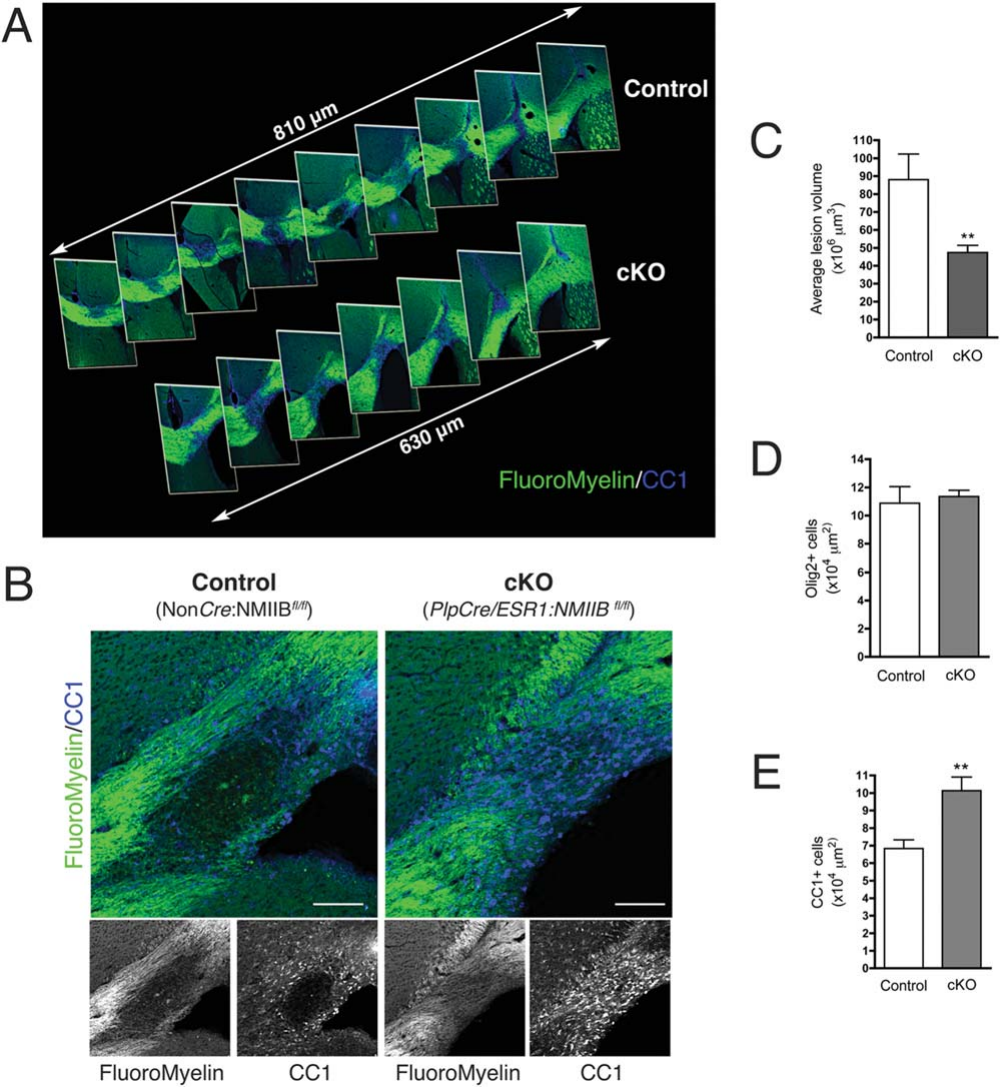
Lesion volume is reduced in mice with conditional ablation of NMIIB. (A): Caudal–rostral (left to right) projection of 30 µm brain sections stained with FluoroMyelin (green) and CC1 antibody (blue) showing a significantly smaller lesion in cKO animals compared with controls at 14 dpi. (B): Images of maximum lesion area at 14 dpi showing an increased number of CC1^+^ OL (blue) in cKO animals compared with controls. Scale bars 100 µm. (C–E): Quantitation of average lesion volume, Olig2+ and CC1^+^ cells at 14 dpi (Mann Whitney *t*-test **P < 0.01). Results were obtained from five to nine animals per genotype, per time point, three different fields per animal.

To test if ablation of NMII in adult brain potentiates CNS remyelination, we induced focal demyelination in 12-week-old cKO (*PlpCre/ESR1: NMIIB ^fl/fl^*), and control (NonCre*:NMIIB ^fl/fl^*) mice, via stereotactic injection of lysolecithin into the corpus callosum. Importantly, analysis 14 days after lysolecithin injection (14 dpi) showed the presence of significantly smaller demyelinated lesions (FluoroMyelin negative area) in the corpus callosum of cKO mice compared with controls (Fig. 3A,B). Quantitation of total lesion volume (Fig. 3C) showed that lesions in the cKO were on average 50% smaller than those found in control animals (47.4 ± 11.2 × 10^6^ µm^3^ for cKO *vs*. 88 ± 40.6 × 10^6^ µm^3^ for control; mean ± SD; *P* = 0.007, *t*-test).

Despite the presence of a comparable number of Olig2+ cells at the lesion site (Fig. 3D) in both control (10.9 ± 1.2 × 10^4^ cells/µm^2^) and cKO mice (11.4 ± 0.4 × 10^4^ cells/µm^2^), we found a significant increase in the number of mature CC1^+^ oligodendrocytes (Fig. 3B,E) present at the lesion at 14 dpi in cKO mice compared with controls (10.1 ± 3 × 10^4^ cells/µm^2^ in cKO *vs*. 6.8 ± 2.2 × 10^4^ cells/µm^2^ in control; mean ± SD; *P* = 0.002, *t*-test). These data suggest that oligodendrocyte differentiation was accelerated in cKO mice, in agreement with our *in vitro* data showing increased OL maturation in cultures derived from NMIIB KO embryonic brains (Wang et al., 2012).

**Figure 4:**
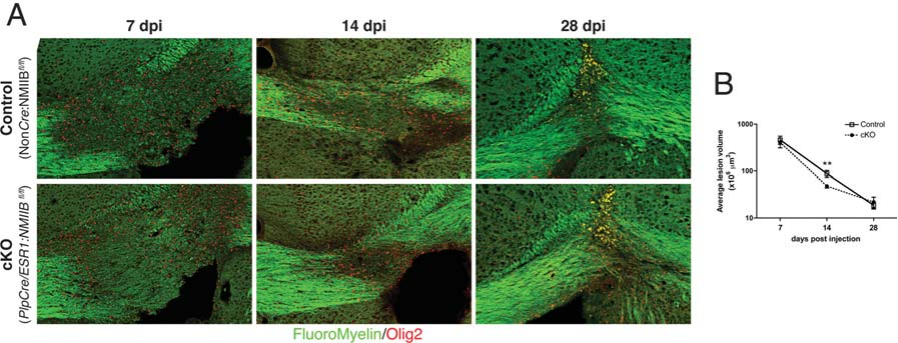
Comparison of lesion progression in control and cKO mice. (A): Fluoromyelin and Olig2 staining of lesion at 7, 14, and 28 days post lysolecithin injection and corresponding lesion volume quantitation (B), showing that the maximal extent of demyelination measured at 7 dpi and lesion resolution at 28 dpi are comparable between the control and cKO mice. Lesion volume only differed significantly at 14 dpi, the peak of remyelination (Mann Whitney *t*-test **P < 0.01). Results were obtained from five to nine animals per genotype, per time point, three different fields per animal.

To rule out that the reduction in lesion size observed in cKO mice at 14 dpi was due to a difference in the initial extent of demyelination, we measured the maximal lesion volume at 7 dpi (Fig. 4A,B), the peak of demyelination in the lysolecithin model, and found that it was comparable to that of controls (338.8 ± 227.1 × 10^6^ µm^3^ for cKO *vs*. 461 ± 181.9 × 10^6^ µm^3^, for controls). Similarly, analysis of lesion volume at 28 dpi (Fig. 4A,B), the end point of the experiment, indicated that both cKO and control mice repaired fully (33.3 ± 12.6 × 10^6^ µm^3^ for cKO *vs*. 19.9 ± 11.7 × 10^6^ µm^3^ for controls).

**Figure 5:**
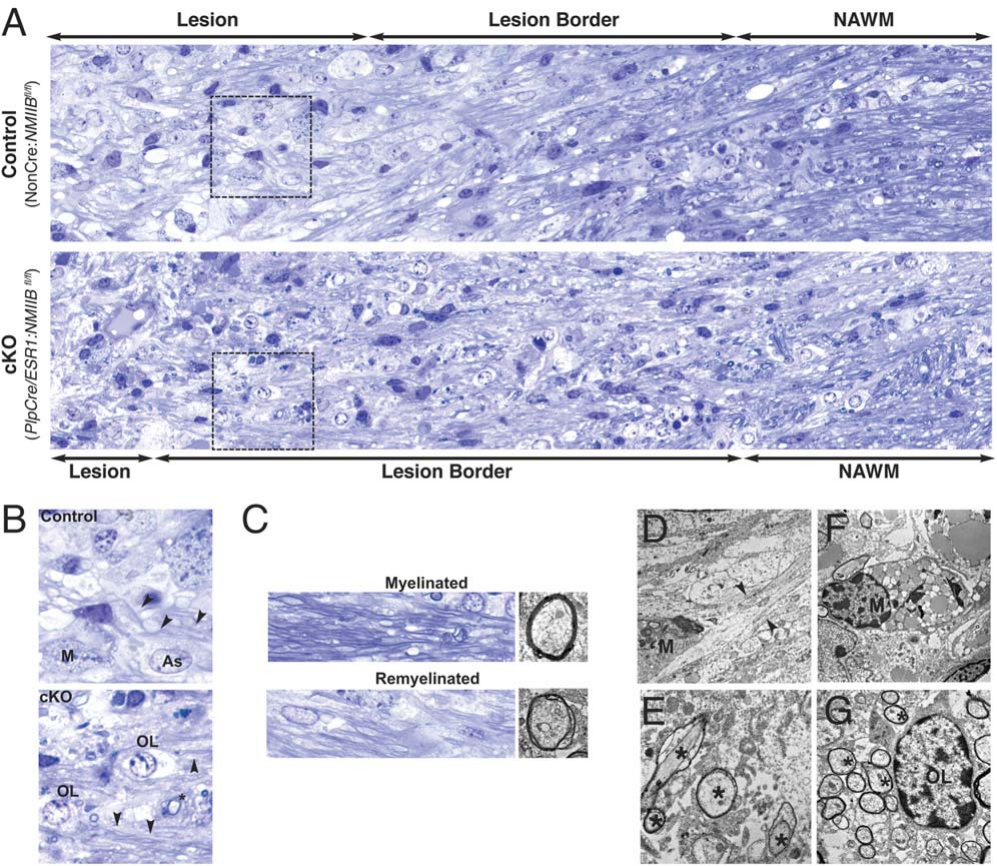
Accelerated remyelination occurs in mice with conditional ablation of NMIIB. (A, B): Light microscopy of epoxy sections stained with toluidine blue showing representative pathology from 14 dpi lysolecithin lesions in control and cKO animals. Normal appearing white matter (NAWM) is also shown. (B): Details of areas boxed in panels (A). The lesion center contains reactive astrocytes (As), lipid-filled mononuclear monocytes (M), and demyelinated axons; but is smaller in cKO animals. The lesion border in cKO contains many axons surrounded by abnormally thin myelin, a characteristic of remyelinated axons (arrowheads). Many cells with morphology consistent with oligodendrocyte lineage (OL) are also observed in the cKO lesion. A remyelinated axon in transverse section (*) is also shown. (C): Examples of normal myelinated axons (top) taken from NAWM area and remyelinated axons (bottom) taken from lesion border area are shown (left panels toluidine blue stain and right panels EM). (D–G): Examples of pathology are illustrated by EM of serial grids from control (D) and cKO (E–G) lesions. (D): A macrophage (M) is seen adjacent to a demyelinated axon (arrowheads) at 7 dpi. (E): Details of multiple remyelinated axons (*) at 7 dpi. (F): A lipid-filled macrophage (M) at 14 dpi. (G): An oligodendroglial cell (OL) is seen at the border of a lesion containing multiple remyelinated axons (*) at 14 dpi.

Morphological analysis by light and electron microscopy (Fig. 5) further confirmed our immunofluorescence results, showing that at 14 dpi, lesions in the cKO brain were smaller (Fig. 5A), and contained more cells with a round, small, and dark nucleus characteristic of oligodendrocytes, as well as many thinly myelinated axons (Fig. 5B,C), a landmark of remyelination (Fields and Ellisman, 1986). Of note, remyelinated axons were observed as early as 7 dpi in cKO (Fig. 5E), a very rare event in control animals (Fig. 5D). Of note, no differences in cell proliferation (Ki67 staining), apoptosis (Caspase 3 staining), or in the total number of Olig2+ at the lesion site were observed between control and cKO lesions (Table1). These data are in agreement with our previous *in vitro* work demonstrating that inhibition of NMII does not enhance proliferation of progenitors in coculture with neurons (Wang et al., 2008), and that in the absence of NMIIB oligodendrocyte differentiation is accelerated as shown by an increase in the number of MBP+ cells isolated from the brains of myosin II KO mice (Wang et al., 2012).

**Table 1:** Comparison of Lysolecithin Lesions at 7 dpi

	Control	cKO
Total DAPI+ cells/area	10.24 ± 0.38 × 10^3^ µm^2^	11.17 ± 0.24 × 10^3^ µm^2^
% Ki67^+^ cells	1.31 ± 0.73%	1.78 ± 0.99%
% Caspase 3^+^cells	0.28 ± 0.05%	0.29 ± 0.06%
% Olig2^+^ cells	9.03 ± 0.05%	8.51 ± 0.75%
NF staining (AU)^a^	61.78 ± 6.13%	61.97 ± 5.16%

Data in table represents mean ± SEM calculated from three to four mice per genotype (three fields per mice).

NF: neurofilament.

a AU: arbitrary units for mean gray value.

Collectively, these results are consistent with the hypothesis that similar to its effects during OL development *in vitro*, ablation NMIIB potentiates myelin formation and repair *in vivo*.

### Axonal Preservation and Recruitment of NG2+ Progenitors Are Not Altered by the Absence of NMIIB

**Figure 6:**
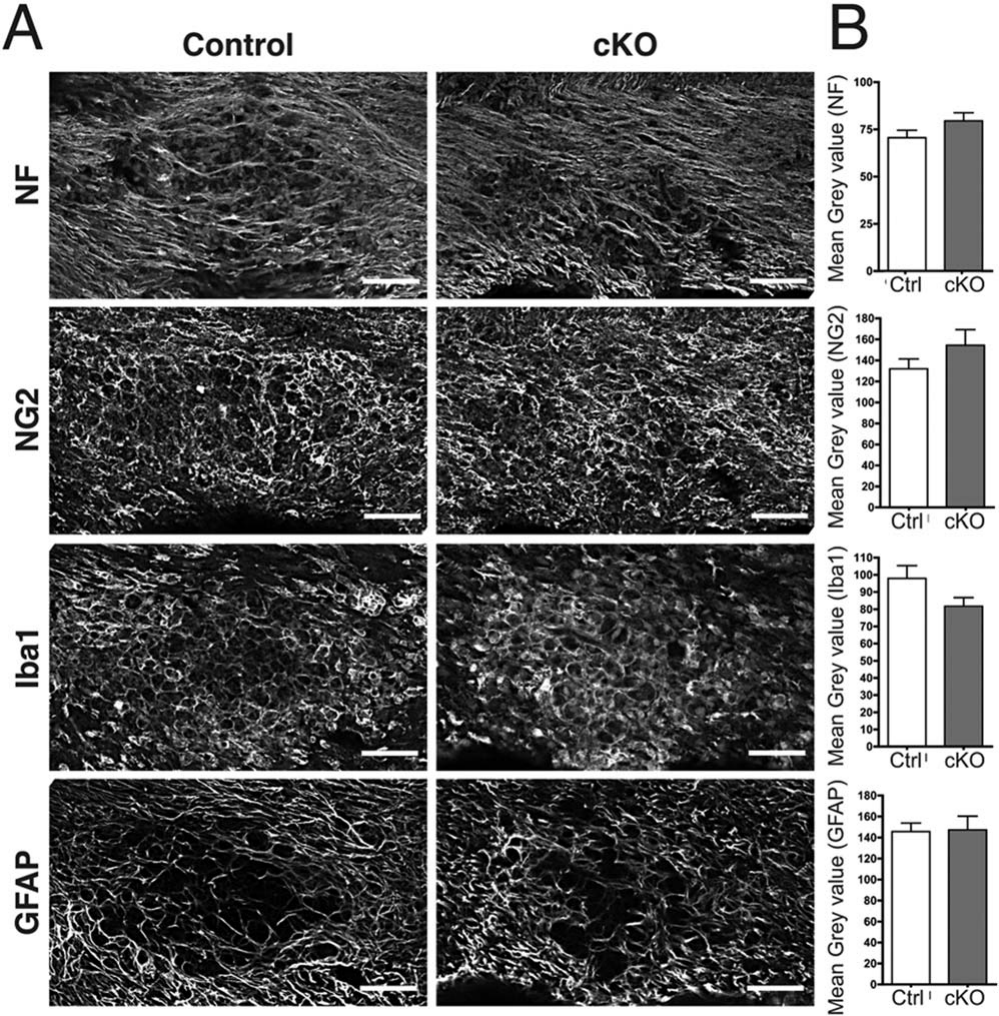
Axonal preservation, progenitor recruitment, inflammation, and reactive astrogliosis are not affected by NMIIB conditional ablation. (A): Sections from control and cKO corpus callosum (14 dpi) immunostained with neurofilament (NF), NG2, Iba1, and GFAP antibodies to evaluate, respectively: axonal preservation, the presence of progenitors, and the extent of inflammation and reactive astrogliosis at the lesion center. (B): Quantitation of fluorescence intensity by normalized mean gray value shows that all parameters were comparable between cKO and control mice, and the small differences in fluorescence intensity were not statistically significant. Data collected from 9 to 10 animals per genotype, three fields per animal. Scale bars 50 µm.

Although primary demyelination is the main effect following focal injection of lysolecithin into white matter, minimal axonal damage has been reported within lesions (Woodruff and Franklin, 1999). As remyelination has been associated with axonal preservation and neuroprotection (Kornek et al., 2000; Raine and Cross, 1989), we examined the extent of neurofilament staining in both cKO and control lesions to establish if axon preservation was enhanced in the absence of NMIIB. We found that neurofilament staining intensity at the center of the lesion was comparable at 7 (Table1) and 14 dpi (Fig. 6) for both control and cKO mice. Although staining appears somewhat reduced in controls compared with cKO at 14 dpi (Fig. 6A,B) this difference was not statistically significant.

Similarly, the staining for NG2+ progenitors, cells that mediate CNS repair and remyelination (Kucharova et al., 2011; Watanabe et al., 2002), although slightly increased in cKO mouse lesions, was not statistically significant compared with controls (Fig. 6A,B). Collectively, these results suggest that improved remyelination in cKO mice was not due to better axonal preservation and/or enhanced recruitment of NG2+ progenitors.

### Inflammation and Reactive Astrogliosis Response at the Lesion Site Are Not Affected by NMII Ablation

Inflammation and astrogliosis are two anticipated responses to local damage, critical for myelin debris clearance and repair (Skripuletz et al., 2013). We investigated whether ablation of NMIIB affected these responses by staining lesions at 14 dpi with antibodies to Iba1, to identify mononuclear phagocytes (macrophages/microglia), and GFAP, to identify astrocytes. Results shown in Fig. 6 indicated that there were no significant differences in the extent of inflammation and astrogliosis between cKO and control animals. Therefore, accelerated repair in the cKO mice could not be attributed to these factors.

### Oligodendrocytes Lacking NMIIB Are Present in Remyelinated Areas

**Figure 7:**
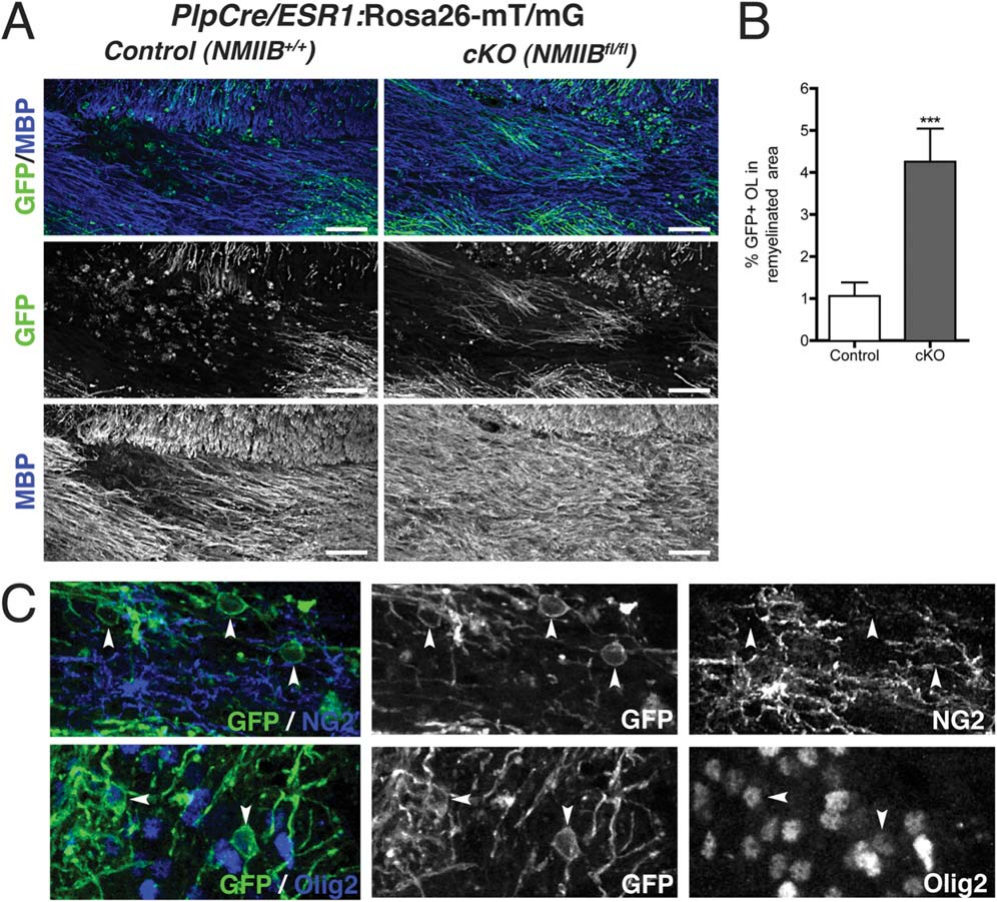
Myelinating oligodendrocytes lacking NMIIB are present in remyelinated areas. (A): Representative images from 28 dpi lysolecithin lesions from transgenic *PlpCre/ESR1*:Rosa26-mT/mG mice carrying wild-type (Control) or floxed (cKO) *NMIIB* alleles. EGFP+ myelinating cells within the remyelinated MBP+ area (blue) were more frequently found in cKO animals. Scale bar 50 µm. (B): Quantitation of percentage area remyelinated by EGFP^+^ OLs in control and cKO animals (Mann Whitney *t*-test ***P < 0.001). Data collected from three animals per genotype, three to five fields per animal. Scale bars 50 µm. (C): Examples of NG2− (top panel); Olig2+ (bottom panel) EGFP-expressing OL (arrowheads) found in the lesion border of remyelinated areas in double transgenic cKO mice.

To investigate if accelerated myelin repair in cKO mice was directly mediated by Plp+ cells lacking NMIIB, we generated double transgenic *PlpCre/ESR1*:Rosa26-mT/mG mice carrying wild-type (Control) or floxed (cKO) NMIIB alleles. Focal demyelination with lysolecithin was induced at 12 weeks as before and lesion resolution was examined at 28 dpi. The results shown in Fig. 7 demonstrate that EGFP+ myelinating OL were more frequently found within the remyelinated area of cKO mice (Fig. 7A). Thus, the percentage of the remyelinated area covered by EGFP+ cells (Fig. 7B) was significantly larger in cKO mice than controls (4.3% ± 2.6 cKO *vs*. 1.06% ± 1.1 control, mean ± SD; *P* = 0.0004, *t*-test).

Double staining of the remyelinated area in the double transgenic cKO mice showed that although NG2+ progenitors predominate in the lesion and lesion border, non-myelinating EGFP+,Olig2+, NG2− cells are also present (Fig. 7C). These cells might represent adult pre-myelinating OL (Chang et al., 2012; Trapp et al., 1997) or surviving demyelinated OL which are known to have a restricted capacity for remyelination in the lysolecithin model (Crang et al., 1998). Importantly, our data indicate that their contribution to myelin repair can be potentiated by NMII ablation.

To test the relevance of our findings to inflammatory-mediated demyelination, we induced focal demyelination by stereotactic injection of pro-inflammatory cytokines (Argaw et al., 2006) in the corpus callosum of control and cKO mice subclinically immunized with MOG. This approach is a modification of cortical EAE (Merkler et al., 2006; Pluchino et al., 2008), a model that mimics human cortical MS lesions and allows the study of the pathogenetic events and remyelination in a temporally well-defined manner. Preliminary results obtained with this model indicate that, similar to our findings with lysolecithin-mediated demyelination, average lesion volume at 15 days post-cytokine injection (the time of lesion resolution in this model) is smaller in cKO mice compared with controls and that GFP+ oligodendrocytes lacking NMIIB contribute to myelin repair (Supp. Info. Fig. S1).

Collectively, these data confirm our previous findings that NMII is a negative regulator of OL development (Wang et al., [Bibr b43],[Bibr b42]); and that its absence/inhibition not only potentiates OL differentiation and *de novo* myelin formation, but it is also relevant to adult myelin repair.

## Discussion

This study demonstrate that downregulation of NMIIB in OL potentiates differentiation and myelin formation *in vivo*, and provides the proof of concept of its relevance to myelin repair. Here, we have performed studies in the context of a toxic insult to myelin, namely lysolecithin-induced demyelination, in the adult mouse corpus callosum. This model permits detailed experimental analysis of the timing and extent of remyelination, a question central to multiple sclerosis (Frohman et al., 2006). By specifically targeting oligodendrocytes using the inducible Cre/lox system driven by the *Plp* promoter, we have been able to demonstrate that mice lacking NMIIB before receiving a demyelinating lesion, which initially exhibits the same size and a similar inflammatory/reactive cell response as those of controls, show accelerated remyelination, and more importantly that Plp-expressing oligodendrocyte-lineage cells contribute to this repair. This result is consistent with our hypothesis that NMIIB acts as negative regulator of OL differentiation (Wang et al., [Bibr b43],[Bibr b42]).

Based on our data, enhanced remyelination in the absence of NMII ablation does not result from differences in proliferation or recruitment of progenitors, nor in enhanced survival as evidenced by Ki67, NG2, and caspase staining and by the presence of similar numbers of Olig2+ cells in both cKO and control lesions. Although we cannot entirely rule out the contribution of non-cell autonomous effects to enhanced remyelination in the cKO brain, we found no evidence at the histological level of changes in the extent of inflammation, astrogliosis and/or axonal damage between cKO and control mice. The only significant differences in the cKO mice were the presence of a smaller lesion area and an increase in the number of CC1+ mature OL at 14 dpi. These changes are consistent with enhanced myelin formation by OL in the absence of NMIIB. In support of this interpretation, we and others have found that inhibition of NMII activity in OL promotes branching, plasma membrane condensation and differentiation (Kippert et al., 2007; Wang et al., 2012), effects that might translate into enhanced myelinogenic potential of individual cells (Chong et al., 2012).

Previous studies have established the participation of SVZ-derived progenitors (Nait-Oumesmar et al., 1999), as well as of NG2+ progenitors (Watanabe et al., 2002), to CNS remyelination after lysolecithin. Despite the presence of large pools of heterogeneous progenitors (Gensert and Goldman, 2001) and the existence of recent studies demonstrating the expression of Plp in a small subpopulation of NG2+ cells in adult brain (Mallon et al., 2002), we observed a significant increase in remyelination by a relatively low number of EGFP+, Olig2+, NG2− oligodendrocytes lacking NMIIB. Thus, using the Rosa26 (mT/mG) reporter we have demonstrated the direct involvement of adult Plp-expressing oligodendrocytes to remyelination. The contribution of this cell population to myelin repair, which are most likely recruited from local sites of myelin injury, has long been debated [reviewed in Bruce et al. (2010)]. Classic studies using transplantation of postmitotic, radiation-resistant cells isolated from adult brain into demyelinated lesions, demonstrated the existence of a heterogenous population of adult oligodendrocytes some of which are capable to form myelin (Crang et al., 1998). Similarly the presence of pre-myelinating oligodendrocyte that can remyelinate axons has also been described (Ludwin, 1979).

As MS targets white matter throughout the CNS (Frohman et al., 2006), affecting regions such as the optic nerve and spinal cord, which are not readily accessible to SVZ-derived progenitors, these disease foci might benefit from strategies that promote remyelination by resident OL. Of note the presence of Plp+, non-myelinating oligodendrocytes, has been reported in adult human and rodent brain as well as in chronic demyelinating lesion in MS (Chang et al., [Bibr b5],[Bibr b4]; Wolswijk, 2000). These cells are likely to reflect a diverse population, which includes mature oligodendrocytes that have lost their myelin and non-myelinating pre-oligodendrocytes. Although the former population is known to have a very restricted remyelination potential (Crang et al., 1998), pre-oligodendrocytes might be stimulated to produce myelin (Ludwin, 1979). Our findings highlight the relevance of this population as potential therapeutic targets for myelin repair.

Previous studies have shown that inhibition of ROCK, a known regulator of NMII activity (Kimura et al., 1996), ameliorates disease progression in mice with EAE (Hou et al., 2012; Huang et al., 2011; Yu et al., 2010). Although the conclusions of these studies implicate modulation of both the inflammatory response and blood–brain barrier permeability as the main cause underlying these beneficial effects, it is not implausible that enhanced differentiation (Kippert et al., 2007; Wang et al., 2012) and OL-mediated remyelination (this work) could also account for improved clinical recovery.

Failure of remyelination may result in axonal atrophy and neurodegeneration, changes that are largely responsible for the progressive functional decline in patients with chronic demyelination. Promotion of endogenous repair may offer the potential to prevent long-lasting damage and clinical disability. Our initial studies using a model of demyelination which mimics some aspects of MS pathology (Supp. Info. Fig. S1) suggest that similar to our findings in the lysolecithin model, repair of demyelinated lesions following inflammatory-mediated damage is also accelerated in the absence of NMII.
