# Supplementary figures and images for: Accelerated repair of demyelinated CNS lesions in the absence of non-muscle myosin IIB
Source: Glia. 2014 Jan 28;62(4):580–91. doi: 10.1002/glia.22627 (PMC4135430; doi:10.1002/glia.22627)

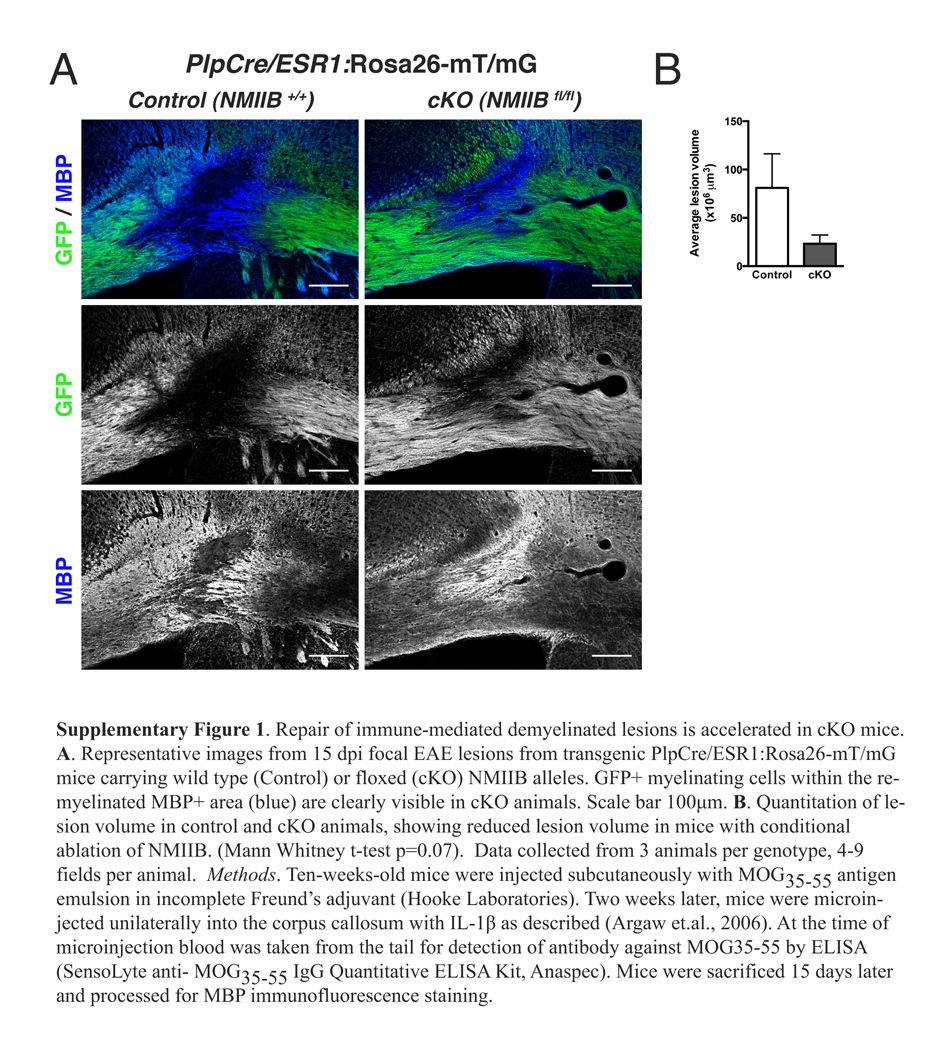

Supplement: Supplementary file 1 — Supplementary Information Figure 1 [file glia0062-0580-sd1.tif]
